# Supplementary material for: Potent latency reversal by Tat RNA-containing nanoparticle enables multi-omic analysis of the HIV-1 reservoir
Source: Nat Commun. 2023 Dec 18;14:8397. doi: 10.1038/s41467-023-44020-5 (PMC10728105; doi:10.1038/s41467-023-44020-5)
Supplement: Supplementary file 1 — Supplementary Information [file 41467_2023_44020_MOESM1_ESM.pdf]

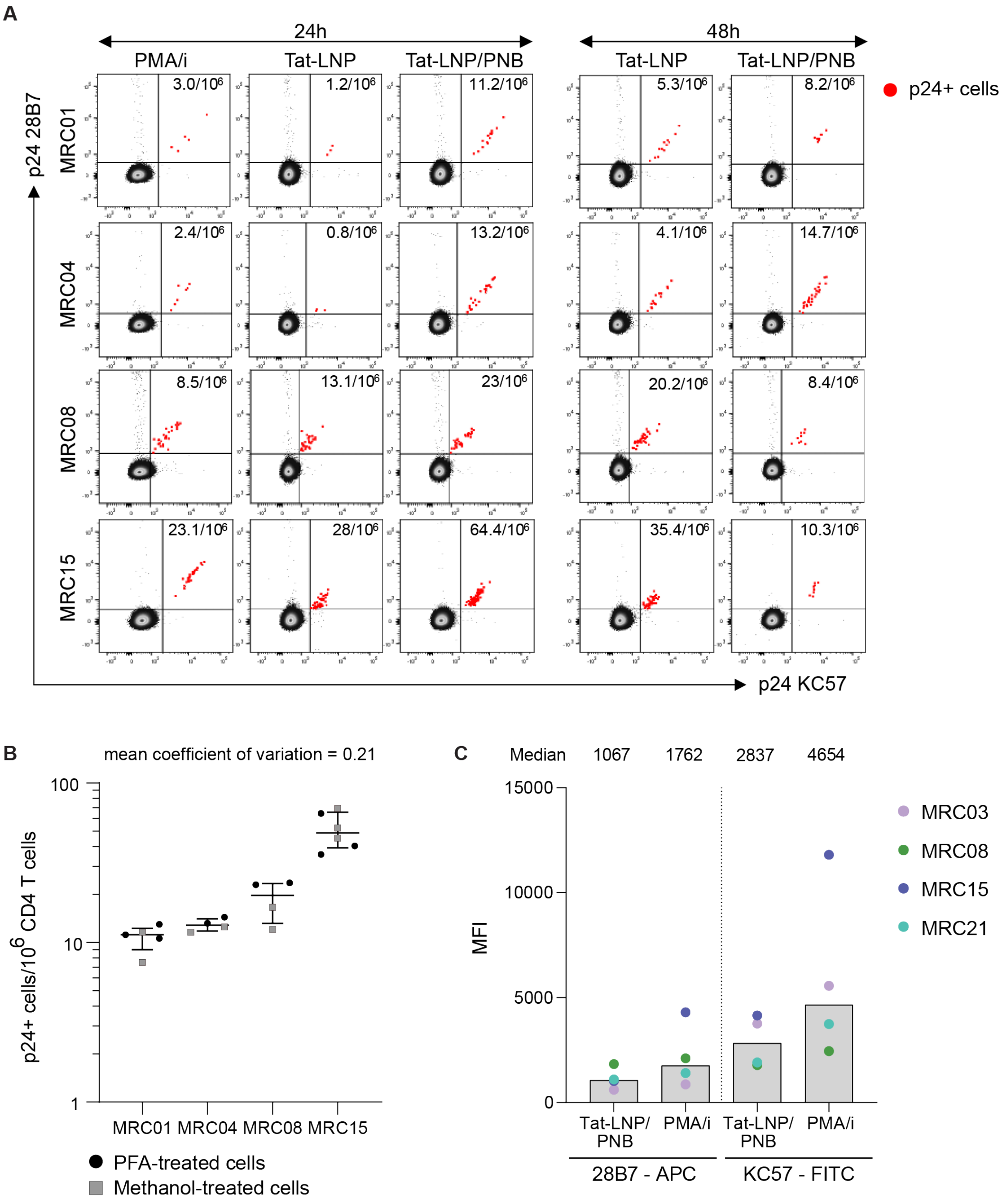


**Supplementary Fig.1. Tat-LNP in combination with PNB induces latency reversal in a higher fraction of cells than PMA/i.** A) FACS dot plots showing the p24 KC57-FITC/28B7-APC co-staining on CD4 T cells from n=4 ART-treated individuals following a 24h or 48h-stimulation with Tat-LNP alone or in combination with PNB. A 24h-stimulation with PMA/i was used as a positive control. P24+ cells are represented in red, and frequencies of p24+ cells are indicated on each dot plot. B) Frequencies of p24+ cells assessed by HIV-Flow in 4-6 individual experiments for each ART-treated participant (n=4). Experiments involving paraformaldehyde (PFA) or methanol fixation are depicted by black circles and grey squares, respectively. C) Median fluorescence intensity (MFI) for the two p24 antibodies (28B7-APC, KC57-FITC) within the p24+ gate following a 24h-stimulation with Tat-LNP/PNB and PMA/i. Participants with a minimal number of 10 p24+ cells in both groups are represented (n=4), and color-coded. Grey bars depict median values. Source data are provided with this paper.

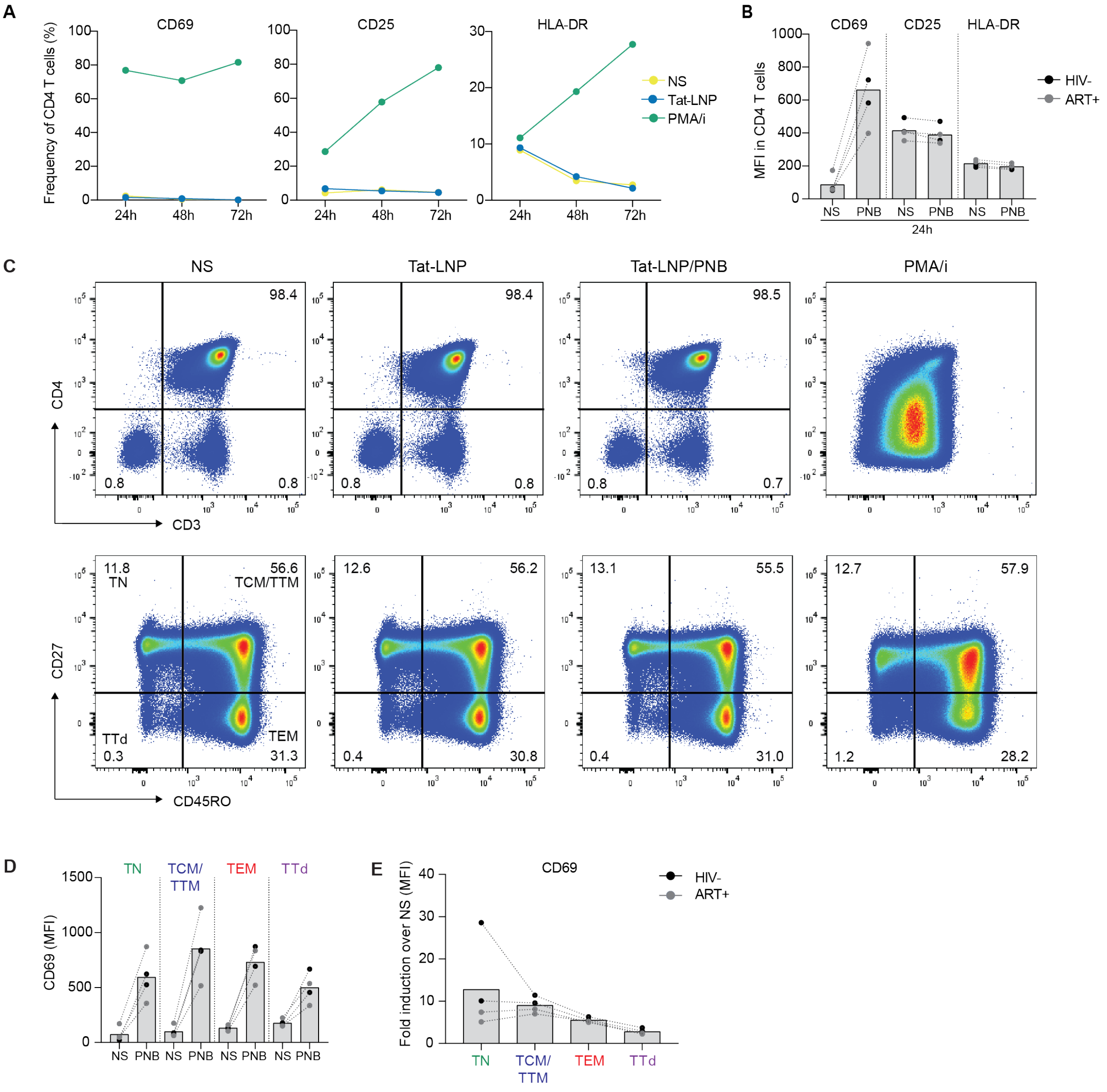

**Supplementary Fig.2. Impact of Tat-LNP and PNB on the phenotype of CD4 T cells.** A) Percentage of cells expressing CD69, CD25 and HLA-DR, as assessed by flow cytometry, in non-stimulated CD4 T cells (NS), or following a 24h, 48h, 72h-stimulation with Tat-LNP and PMA/i. B) Mean fluorescence intensities (MFI) of CD69, CD25 and HLA-DR are compared between non-stimulated (NS) and PNB-stimulated cells within bulk CD4 T cells (n=2 HIV- controls and 2 ART-treated individuals). C) Representative FACS dot plots showing the CD3/CD4 and CD45RO/CD27 co-stainings following a 24h-stimulation with Tat-LNP, Tat-LNP/PNB and PMA/i. Proportions of each subset are indicated on each dot plot. D) CD69 MFI are compared between non-stimulated (NS) and PNB-stimulated cells within CD4 T cell subsets (n=2 HIV- controls and 2 ART-treated individuals). E) Box plots showing CD69 MFI, expressed as a fold induction over the NS condition, within T cell subsets. Grey bars depict mean values. TN = naïve T cells, TCM/TTM = central and transitional memory T cells, TEM = effector memory T cells, TTd= terminally differentiated T cells. Source data are provided with this paper.

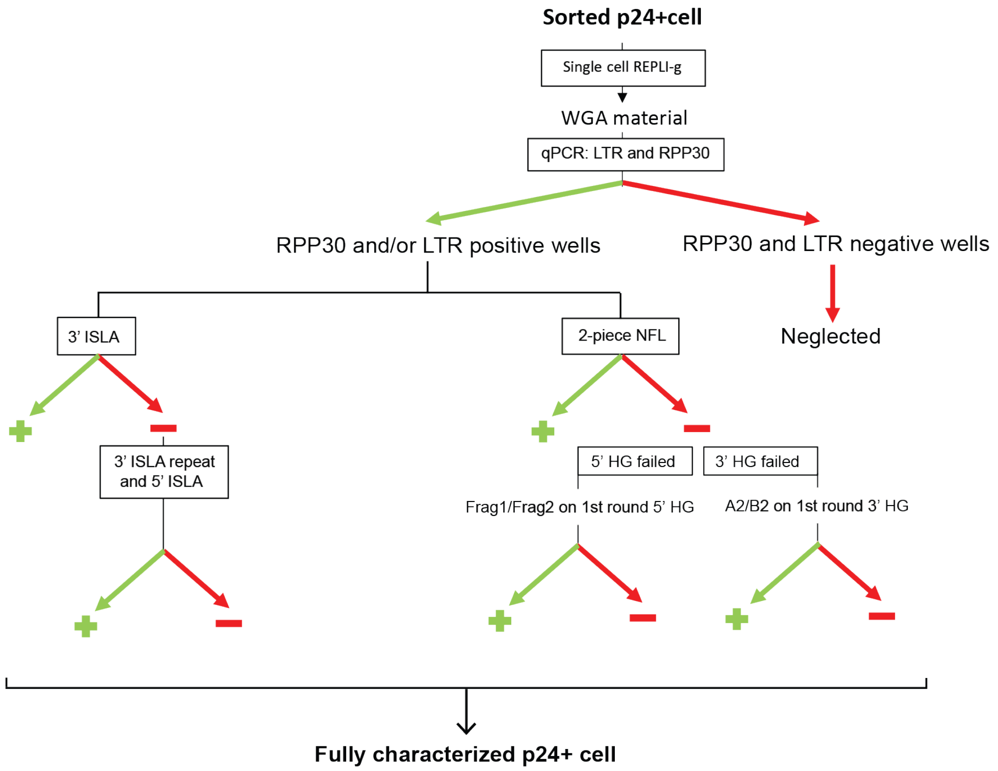


**Supplementary Fig.3. STIP-Seq decision tree.** Step-by-step overview of the methodologies applied during STIP-Seq. Single sorted p24+ cells are subjected to whole genome amplification by multiple displacement amplification. Reactions are screened for successful amplification with a duplex qPCR (RPP30 reference gene, HIV LTR). Wells containing a cell that is RPP30 and/or LTR positive by qPCR are subjected to integration site analysis by integration site loop amplification (ISLA) and near full-length proviral sequencing with a 2- amplicon approach (5' and 3' half genomes). In the case of cells that failed to retrieve a band on gel for the 5' amplicon: the first-round material from the 5' amplicon is subjected to 2 PCRs (Frag1/Frag2). In the case of cells that failed to retrieve a band on gel for the 3' amplicon: the first-round material from the 3' amplicon is subjected to 2 PCR (A2/B2). The list of primers and probes used is shown in Supplementary Table 2. WGA = whole genome amplification, LTR = long terminal repeat, ISLA = Integration Site Loop Amplification, NFL = Near full-length sequencing, HG = half genome.

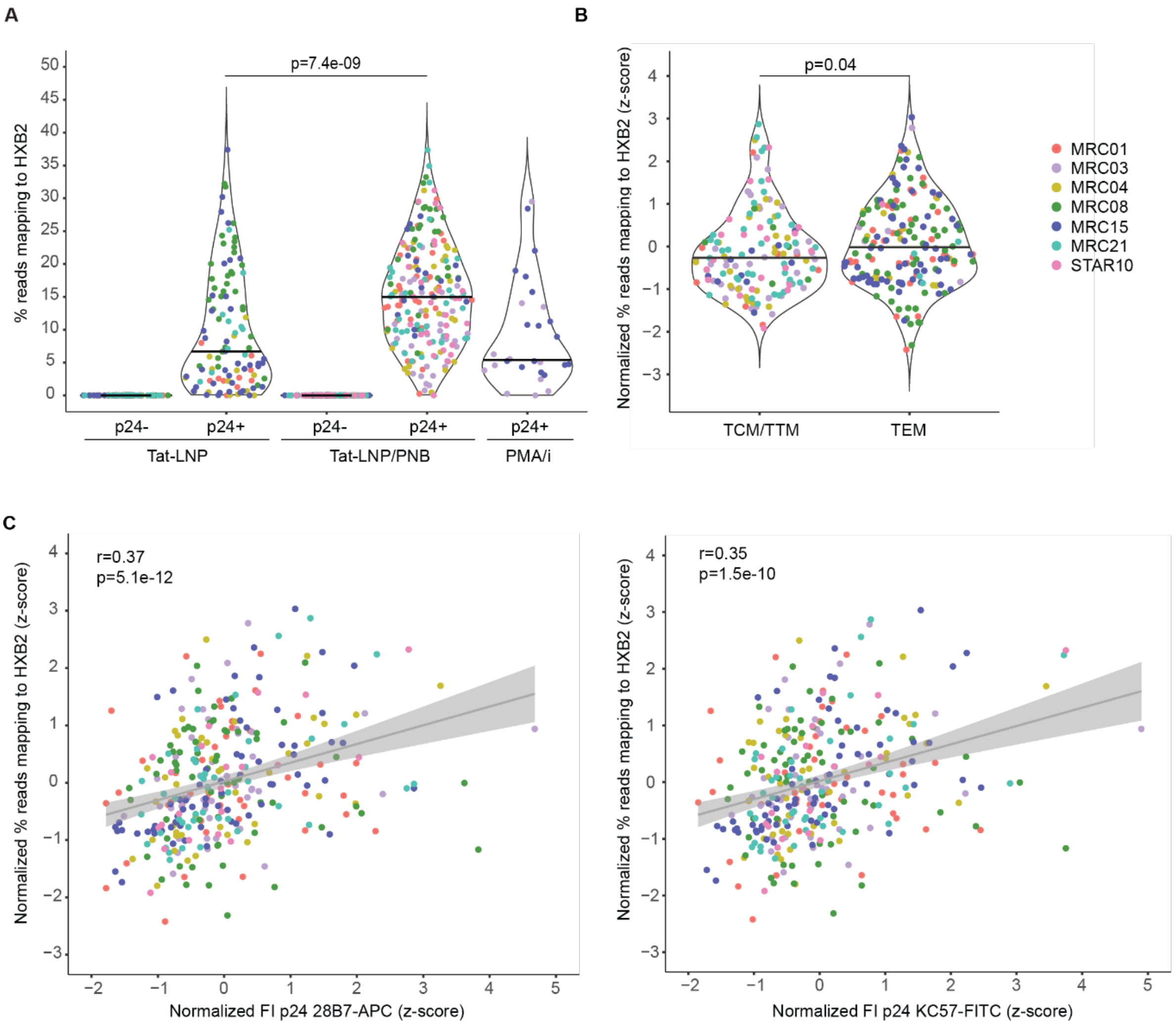


**Supplementary Fig.4. The percentage of HIV reads correlates with the p24 fluorescence intensity.** A) Violin plots showing the percentage of reads mapping to the reference genome HXB2 in p24- versus p24+ cells following stimulation with Tat-LNP alone (48h), combined to PNB (24h), or following PMA/i stimulation (24h). Each dot represents a single cell processed by Smart-seq2, black horizontal bars depict median values, and participants are color-coded. A non-parametric Mann-Whitney test was used for statistical analysis. B) Violin plots depicting the normalized percentage of reads mapping to HXB2 in the TCM/TTM and TEM subsets. All treatment conditions (Tat-LNP, Tat-LNP/PNB, PMA/i) are combined for this analysis. Black horizontal bars depict the median values. A non-parametric Mann-Whitney test was used to compare both groups. TCM/TTM = central and transitional memory T cells, TEM = effector memory T cells. C) Correlations between the normalized percentage of reads mapping to HXB2 and the normalized fluorescence intensity (FI) for p24 28B7-APC (left) and for p24 KC57-FITC (right). Each dot represents a sorted p24+ cell analyzed by Smart-seq2. The FI was analyzed by index cell sorting. The FI and the percentage of HIV reads were normalized by z-score for each experiment (normalized FI = [FI - mean(FI from all p24+ cells)] / SD(FI from all p24+ cells)). Participants are color-coded. Non-parametric Spearman rank correlation tests were performed. Source data are provided with this paper.

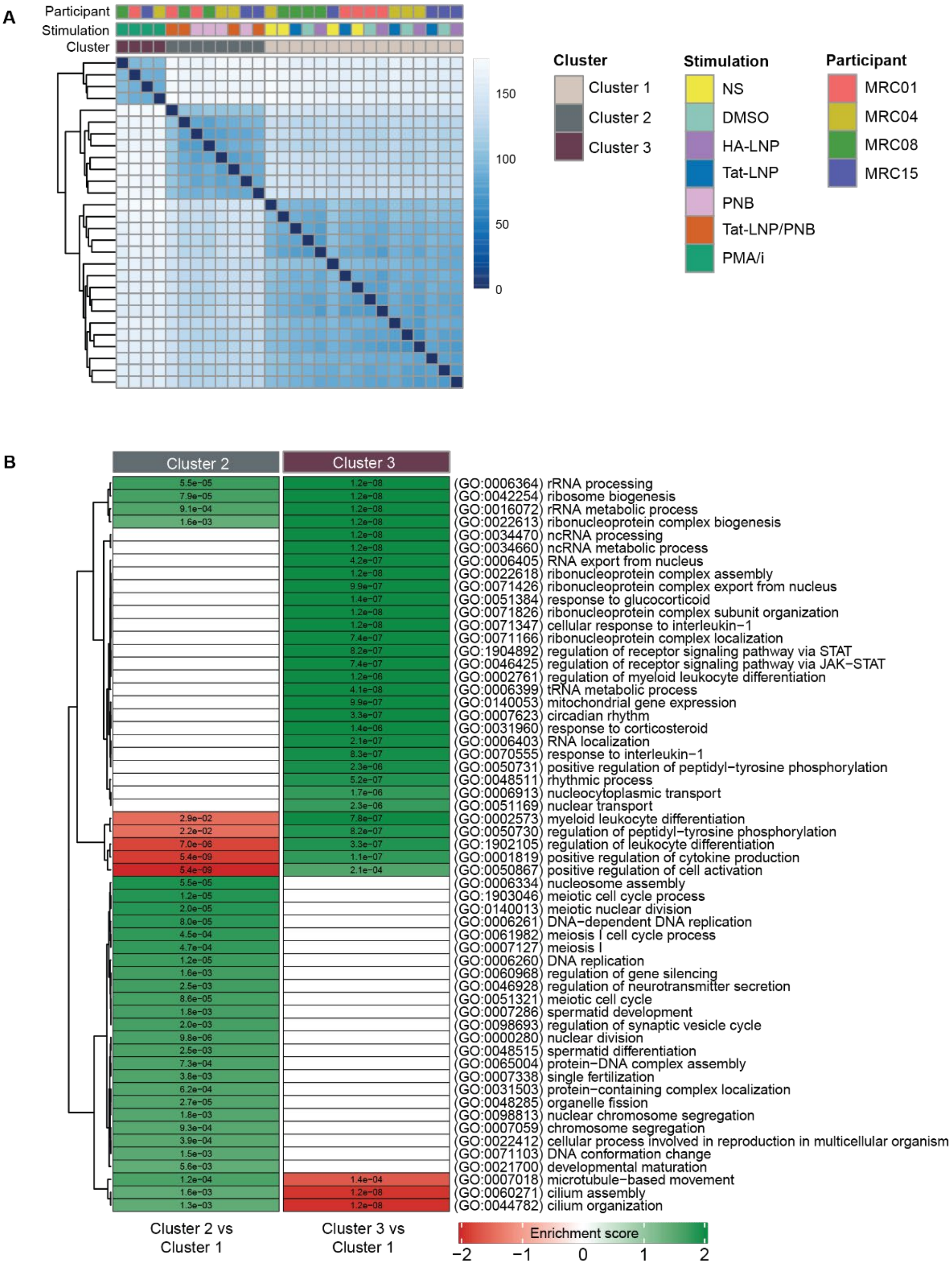


**Supplementary Fig.5. Tat-LNP does not modify the transcriptome of CD4 T cells**. A) Bulk CD4 T cells were stimulated for 6h with the LRAs of interest and micro-array analyses were performed. Correlation matrix identifying 3 separate clusters based on cell stimulation: cluster 1) NS, DMSO, HA-LNP, Tat-LNP; cluster 2) PNB and Tat-LNP/PNB, cluster 3) PMA/i. Clusters, participants and stimulation conditions are color-coded and represented on the top of the correlation matrix. The dendrogram shows the hierarchical relationship between samples. The color scale shows the value of the correlation between 2 samples (Euclidean distance): dark blue highlights strong correlation values, while light blue highlights weak correlation values. B) Gene Set Enrichment Analysis (GSEA) analysis comparing cluster 2 (PNB, Tat-LNP/PNB) and cluster 3 (PMA/i) to cluster 1 (NS, DMSO, HA-LNP, Tat-LNP). The color scale is showing the enrichment score for each GO term: datasets represented in green contain genes that are upregulated in the cluster of interest compared to cluster 1, datasets represented in red contains genes that are downregulated in the cluster of interest compared to cluster 1. P-values are indicated on the graphs. The lists of differentially expressed genes and pathways between clusters are shown in Supplementary Data 3 (6h of stimulation) and 4 (24h of stimulation).

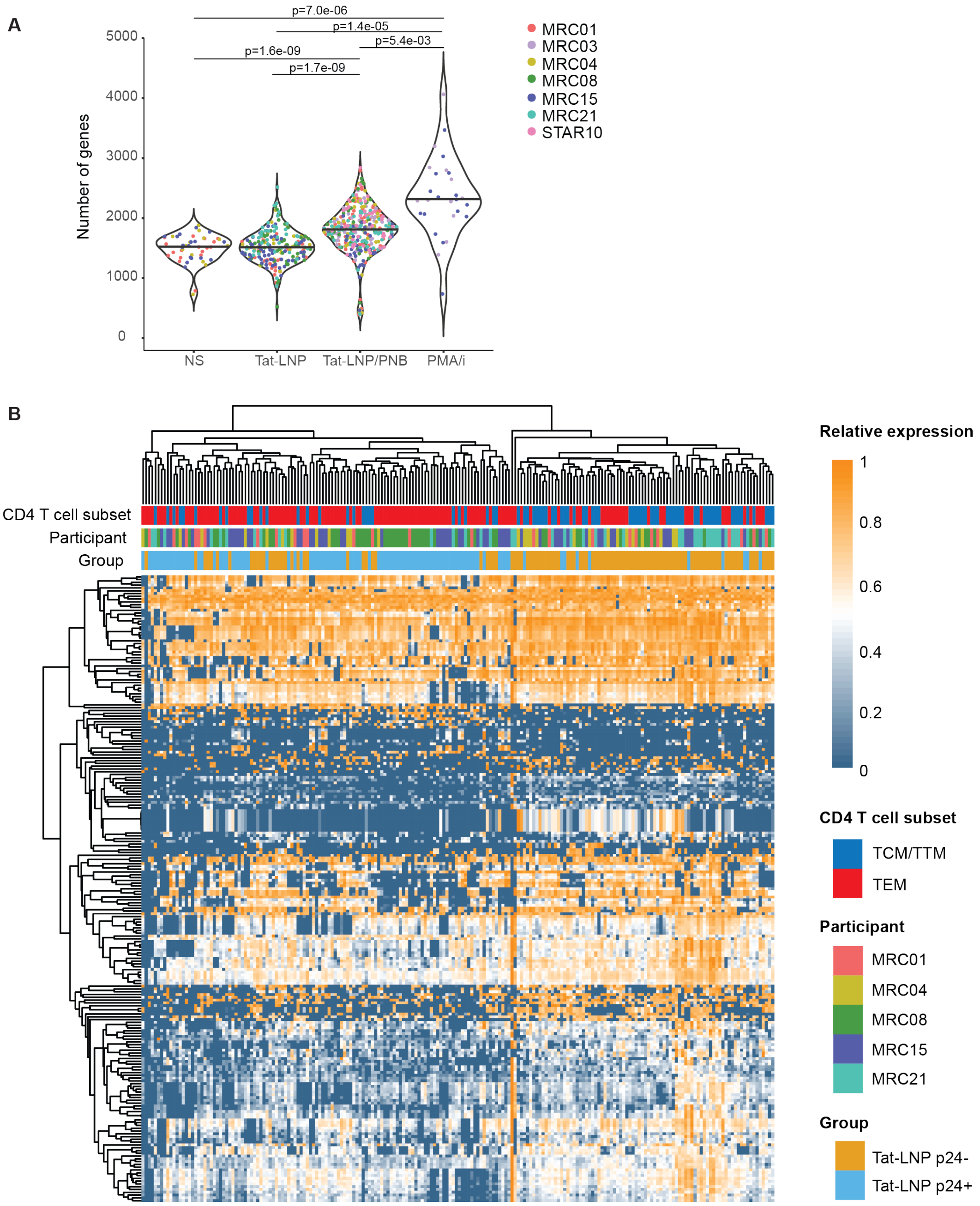


**Supplementary Fig.6. P24+ cells display a distinct transcriptional landscape compared to p24- cells.** A) Violin plots showing the number of genes detected per p24+ cell following Smart-seq2 processing. Four groups are represented based on stimulation: non-stimulated (NS) cells (48h), Tat-LNP (48h), Tat-LNP/PNB (24h) and PMA/i (24h)-stimulated cells. Each dot represents a single cell processed by Smart-seq2, black horizontal bars depict median values, and participants are color-coded. For pairwise comparisons between groups, the Games Howell test with Bonferroni adjustment for multiple testing was used. Only significant adjusted p-values ($p<0.05$) are shown on the graph. B) Heatmap showing the unsupervised clustering of the differentially expressed genes between p24+ and p24- cells following Tat-LNP stimulation (48h). CD4 T cells subsets, participants, and p24 expression are color-coded. The scale bar indicates the relative expression of each differentially expressed gene for each individual cell. Source data are provided with this paper.

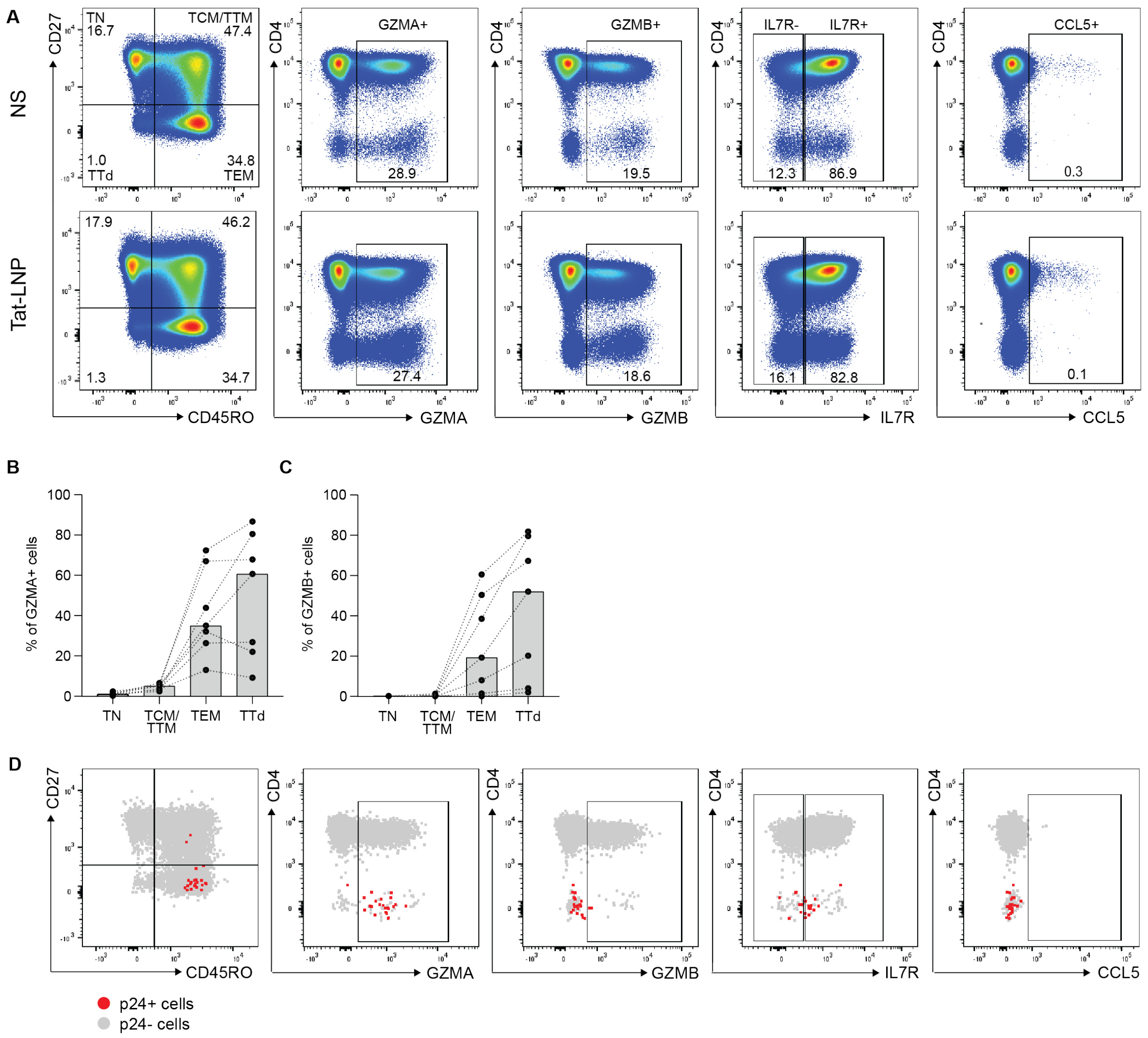


**Supplementary Fig.7. Transcriptomic differences between p24+ and p24- cells are confirmed at the protein level.** A) Representative FACS dot plots showing CD4 T cell subsets (TN, TCM/TTM, TEM, TTd) as well as the expression of GZMA, GZMB, IL7R and CCL5 in absence of stimulation (upper panel) or following a 48h-treatment with Tat-LNP (lower panel). Proportions of each subset are indicated on each dot plot. (B-C) Percentage of cells expressing GZMA (B) and GZMB (C) in each CD4 T cell subset. Grey bars depict median values. D) Representative FACS dot plots showing the phenotype of p24+ cells (in red) overlaid to p24- cells (in grey). Gated on LiveDead-/CD8-/CD3+ cells. TN = naïve T cells, TCM/TTM = central and transitional memory T cells, TEM = effector memory T cells, TTd= terminally differentiated T cells. Source data are provided with this paper.

| Participant ID | Age | Gender | CD4/CD8 ratio | NADIR | Subtype | VL | Time since infection | Time to ART | ART duration | HIV-Flow | STIP-Seq | scRNA-seq |
|---|---|---|---|---|---|---|---|---|---|---|---|---|
| MRC01 | 50-60 | M | 0.3 | 44 | B | <20 | NA | NA | 18.5 | x | x | x |
| MRC02 | 60-70 | M | 1.2 | 484 | B | <20 | NA | NA | 4.4 | x | | |
| MRC03 | 30-40 | M | 0.7 | 114 | F1 | <20 | 30.7 | 16.0 | 14.6 | x | | x |
| MRC04 | 50-60 | M | 1.0 | 171 | B | <20 | NA | NA | 18.4 | x | x | x |
| MRC05 | 40-50 | M | 1.3 | 492 | B | <20 | 13.0 | 6.9 | 6.1 | x | | |
| MRC06 | 50-60 | F | 0.9 | 226 | C | <20 | NA | NA | 3.7 | x | | |
| MRC07 | 30-40 | M | 1.1 | 382 | B | <20 | 7.5 | 2.4 | 5.1 | x | | |
| MRC08 | 50-60 | M | 0.5 | 488 | B | <20 | 16.5 | 15.2 | 1.3 | x | x | x |
| MRC09 | 50-60 | F | 0.8 | 102 | A1 | <20 | NA | NA | 17.1 | x | | |
| MRC11 | 30-40 | M | 1.3 | 350 | B | <20 | 8.4 | 1.1 | 7.3 | x | | |
| MRC12 | 30-40 | M | 1.0 | NA | B | <20 | 11.7 | 4.2 | 7.5 | x | | |
| MRC13 | 20-30 | M | 1.3 | 601 | Recomb B/F1 | <20 | NA | NA | 7.2 | x | | |
| MRC14 | 60-70 | M | 0.9 | 211 | CRF02_AG | <20 | 11.0 | 5.1 | 5.9 | x | | |
| MRC15 | 50-60 | M | 0.6 | 98 | B | <20 | NA | NA | 14.7 | x | x | x |
| MRC19 | 30-40 | M | 0.8 | 395 | B | <20 | NA | NA | 1.4 | x | | |
| MRC20 | 40-50 | M | 0.6 | 294 | B | <20 | 19.8 | 3.4 | 16.4 | x | | |
| MRC21 | 60-70 | M | 0.9 | 179 | B | <20 | 30.6 | 5.8 | 24.8 | x | x | x |
| MRC22 | 60-70 | M | 0.8 | 196 | B | <20 | 11.6 | 3.0 | 8.7 | x | | |
| MRC23 | 50-60 | M | 0.8 | 182 | B | <20 | 17.0 | 0.7 | 16.3 | x | | |
| MRC24 | 50-60 | M | 0.9 | 231 | B | <20 | 15.6 | 2.8 | 12.8 | x | | |
| MRC25 | 40-50 | M | 0.7 | 361 | B | <20 | 14.1 | 2.6 | 11.5 | x | | |
| UZG3034 | 30-40 | M | 0.7 | 356 | B | <20 | 3.0 | 0.5 | 2.4 | x | | |
| STAR10 | 50-60 | M | 0.7 | 327 | B | <20 | 21.3 | 3.0 | 18.3 | x | x | x |
| Median | 50.3 | | 0.8 | 262.5 | | | | | 8.7 | | | |
| IQR | 39.2-55.6 | | 0.7-1 | 179.8-376.8 | | | | | 5.5-16.4 | | | |

**Supplementary Table 1. Clinical characteristics of the participants.**

| Participant ID | Total HIV DNA (cps/$10^6$ CD4 T cells) | Frequency of p24+ cells (per $10^6$ CD4 T cells) | | Frequency of p24+ cells/total HIV DNA (%) | |
|---|---|---|---|---|---|
| | | Tat-LNP | Tat-LNP/PNB | Tat-LNP | Tat-LNP/PNB |
| MRC01 | 2592.3 | 2.0 | 12.7 | 0.1 | 0.5 |
| MRC04 | 779.8 | 2.9 | 10.5 | 0.4 | 1.3 |
| MRC08 | 450.5 | 28.5 | 15.7 | 6.3 | 3.5 |
| MRC15 | 183.6 | 33.3 | 52.4 | 18.1 | 28.5 |
| MRC21 | 5690.9 | 6.3 | 33.0 | 0.1 | 0.6 |

**Supplementary Table 2. Assessment of the size of the total and translation-competent viral reservoir.** Total HIV DNA was measured by digital PCR in CD4 T cells targeting the LTR region of HIV; RPP30 was used as reference gene. The frequency of p24+ cells following Tat-LNP and Tat-LNP/PNB treatments was measured by the HIV-Flow assay. For each participant, the proportion of the total reservoir that is reactivated by Tat-LNP or Tat-LNP/PNB is displayed on the two last columns.

| Assay | STIM | SORT | Intactness | Transcriptome | Proteome | Main discovery |
|---|---|---|---|---|---|---|
| **HIV-infected cells (HIV DNA+ cells)** | | | | | | |
| **TCR-based** Weymar et al, Cell Reports, 2022 | NO | Clones with intact proviruses based on their unique TCR | Assessed prior sorting | YES | NO | HIV+ CD4 T cell clones carrying intact proviruses preferentially express HLA-DR, HLA-DP, CD74, **CCL5**, **GZMA** and GZMK, cystatin F, LYAR, and DUSP2. |
| **FIND-seq** Clark et al, Nature, 2023 | NO | NO | NO | YES | NO | HIV+ memory CD4 T cells from ART-treated individuals display signatures of HIV silencing, cell survival and cell proliferation. |
| **ASAP-seq** Wu et al, Nat Immunol, 2023 | NO | NO | NO | NO | ADT | HIV+ CD4 T cells from ART-treated individuals display significantly higher expression of CCR5, PD-1 and CD2 compared to HIV- cells. |
| **PheP-seq** Sun et al, Nature, 2023 | NO | NO | +/- | NO | ADT | HIV+ CD4 T cells from ART-treated individuals show only slight phenotypic variations relative to HIV- cells. HIV+ cells with an intact provirus display significantly higher expression of PD1, TIGIT, BTLA, 2B4, KLRG1, CD49d, CD127, IL-21R, TGFβR relative to HIV- cells. |
| **Transcription-competent reservoir (HIV RNA+ cells)** | | | | | | |
| **SortSeq** Liu et al, STM, 2020 | PMA/i | HIV RNA+ cells | NO | YES | NO | HIV-1 SortSeq+ cells significantly up-regulate IMPDH1, JAK1, IKBKB, IL2, LTA, CCL3, CCL4, XCL1, and reveal enrichment of nonsense-mediated RNA decay and viral transcription pathways relative to HIV SortSeq- cells. |
| **ECCITE-seq** Collora et al, Immunity, 2022 | NO | NO | NO | YES | ADT | HIV-1 RNA+ T cell clones upregulate cytotoxic T cell genes (GZMB, GZMH, **GZMA**, PFN1, **CCL5**, CST7, NKG7) and IL2RG relative to HIV-1 RNA– T cell clones in untreated individuals. Protein-based validations using flow cytometry confirm those data in ART-treated individuals. |
| **Translation-competent reservoir (HIV proteins+ cells)** | | | | | | |
| **LURE** Cohn et al, Nat Med, 2018 | PHA | Env+ cells | NO | YES | NO | Env+ cells significantly upregulate TIGIT, HLA-DR, miR155, CCL3, PRDM1, and suppress pathways associated with cellular anti-viral immunity. |
| **HIV-Flow** Pardons et al, Plos Path, 2019 | PMA/i | NO | NO | NO | Flow cytometry | P24+ cells significantly upregulate immune checkpoint molecules (PD-1, TIGIT) and the integrin α4β1 relative to p24- cells. |
| **CyTOF** Neidleman et al, eLife, 2020 | PMA/i | NO | NO | NO | Mass spectrometry | P24+ cells significantly upregulate immune checkpoint (PD-1, CTLA4), activation (CD69, CD25, HLADR), and T cell differentiation markers (Tbet, CRTH2, CCR6). |
| **Smart-seq2 on p24-/p24+ cells** | Tat-LNP | p24+ cells | +/- | YES | Flow cytometry | P24+ cells significantly upregulate a novel long non-coding RNA LINC02964, GZMA, CCL5, while downregulating ATG10 and IL7R. P24+ cells exhibit heightened PI3K/Akt signaling, along with downregulation of protein translation |

ADT = Antibody-Derived Tags

**Supplementary Table 3. Review of recent single-cell RNA-seq studies analyzing the phenotype of HIV-infected cells.**

| Assay | Amplicon | HXB2 coordinates | Forward/Reverse | Round | Primer | Sequence (5' to 3') |
|---|---|---|---|---|---|---|
| RPP30 qPCR | RPP30 | | Forward | 1 | Forward | AGATTTGGACCTGCGAGCG |
| | | | Reverse | 1 | Reverse | GAGCGGCTGTCTCCACAAGT |
| | | | Probe | 1 | Probe | TTCTGACCTGAAGGCTCTGCGCG |
| ISLA 3'end | | 8948 | Forward | 1 | up3.2 | CCAATGCTGATTGTGCCTGGCTAGAAGCA |
| | | NA | Forward | 2 | deca1.u5 | TCAAGTAGTGTGTGCCCGTCTGTNNNNNNNNNN |
| | | 9553 | Forward | 3 | RF2 | AGACCAGATCTGAGCCTGGGAGCTCTCTG |
| | | 9595 | Forward | 4 | RF1 | CCCACTGCTTAAGCCTCAATAAAGCTTGCCTTG |
| | | 9626 | Forward | 5 | 1.U5 | TGAGTGCTTCAAGTAGTGTGTGCCCGTCTGT |
| | | 9647 | Forward | 6 | 2.U5 | GCCCGTCTGTTGTGTGACTCTGGTAACTAGAGAT |
| ISLA 5'end | | 651 | Reverse | 1 | UTR.629.R | CCCTGTTCGGGCGCCACTGCTA |
| | | NA | Reverse | 2 | decaU3R.3 | GTTCTGCCAATCAGGGAAGTAGCCTTGTGTGTNNNNNNNNNN |
| | | 160 | Reverse | 3 | U3R.1 | GGCTCAACTGGTACTAGCTTGAAGCACCATCCAAAG |
| | | 118 | Reverse | 4 | U3R.2 | GGATATCTGATCCCTGGCCCTGGTGTGTAGTT |
| | | 89 | Reverse | 5 | U3R.3 | GTTCTGCCAATCAGGGAAGTAGCCTTGTGTGT |
| | | 51 | Reverse | 6 | U3R.4 | CCCACAGATCAAGGATATCTTGTCT |
| 2-amplicon NFL | Left half (5') | 544-5968 | Forward | 1 | F544 | TTAAGCCTCAATAAAGCTTGCCTTGAG |
| | | | Reverse | 1 | R5968 | TGTCTYCKCTTCTTCCTGCCATAG |
| | | 581-5783 | Forward | 2 | F581 | GTGTGCCCGTCTGTTGTGTGACTC |
| | | | Reverse | 2 | R5783 | AATGCCTATTCTGCTATGTYGACACC |
| | Right half (3') | 5066-9665 | Forward | 1 | F5066alt1 | TATGGAAAACAGATGGCAGGTGMTGRT |
| | | | Reverse | 1 | R9665 | GTCTGAGGGATCTCTAGWTACCAGA |
| | | 5088-9602 | Forward | 2 | F5088alt1 | GATTGTGTGGCARGTAGACAGRATG |
| | | | Reverse | 2 | R9602 | CAAGGCAAGCTTTATTGAGGCTTAAS |
| NFL primers described by Einkauf et al, J Clin Invest, 2019 | A2 | 5549-7760 | Forward | 2 | VP5549F | AGAGGATAGATGGAACAAGCCCCAG |
| | | | Reverse | 2 | V3CR | TGCTCTTTTTTCTCTCTSCACCACT |
| | B2 | 7652-9610 | Forward | 2 | GP41Fi | GGACAATTGGAGAAGTGAATTAT |
| | | | Reverse | 2 | 3UTRi | AGGCTTAAGCAGTGGGTTCCCTAG |
| NFL primers described by Patro et al, PNAS, 2019 | Frag 1 | 634-3500 | Forward | 2 | 634(+) | AGTGGCGCCCGAACAGGGAC |
| | | | Reverse | 2 | 3500(-) | CTATTAAGTATTTTGATGGGTCATAA |
| | Frag 2 | 1870-5248 | Forward | 2 | 1870(+) | GAGTTTTGGCTGAGGCAATGAG |
| | | | Reverse | 2 | 5248(-) | TCTCCTGTATGCAGACCCCA |
| Smart-seq2 | | | | | TSO | AAGCAGTGGTATCAACGCAGAGTACATrGrG+G |
| | | | | | Oligo-dT30VN | AAGCAGTGGTATCAACGCAGAGTACT30VN |
| | | | | | ISPCR oligo | AAGCAGTGGTATCAACGCAGAGT |
| Reference genes qPCR | ACTB | | Forward | | Forward | TTCCTTCCTGGGCATGGAGT |
| | | | Reverse | | Reverse | TACAGGTCTTTGCGGATGTC |
| | GAPDH | | Forward | | Forward | AGCCTCAAGATCATCAGCAATGCC |
| | | | Reverse | | Reverse | TGTGGTCATGAGTCCTTCCACGAT |
| | YHWHAZ | | Forward | | Forward | ACTTTTGGTACATTGTGGCTTCAA |
| | | | Reverse | | Reverse | CCGCCAGGACAAACCAGTAT |
| | β2M | | Forward | | Forward | AGATGAGTATGCCTGCCGTGTGAA |
| | | | Reverse | | Reverse | TGCTGCTTACATGTCTCGATCCCA |
| lncRNA qPCR | LINC02964 | | Forward | | Forward | GTCTGGTGGAAAAGGATCATG |
| | | | Reverse | | Reverse | AGACATCTGCAACTCCTGACTC |

**Supplementary Table 4. List of primers used in this manuscript.** NFL = near full-length proviral sequencing, ISLA = integration site loop amplification.

| | Number of genes per cell | | | Number of mapped reads per cell | | | Percentage of mitochondrial reads per cell | | |
|---|---|---|---|---|---|---|---|---|---|
| | All | p24+ | p24- | All | p24+ | p24- | All | p24+ | p24- |
| Median | 1691 | 1690 | 1746 | 988151 | 952517 | 1018455 | 8.8711 | 9.3718 | 8.2065 |
| IQR | 1474-1929 | 1476-1977 | 1491-1956 | 848924-1191324 | 802911-1180938 | 883220-1196057 | 7.1794-10.7666 | 7.7063-11.3812 | 6.7527-10.0592 |
| Minimum | 322 | 412 | 322 | 645 | 42178 | 645 | 0.3101 | 0.0028 | 0.3101 |
| Maximum | 4063 | 4063 | 2840 | 2601259 | 2601259 | 2327991 | 43.2573 | 17.3576 | 43.2573 |

**Supplementary Table 5. QC metrics from single-sorted cells processed by Smart-seq2.** Number of genes per cell, number of mapped reads per cell, and the percentage of mitochondrial reads per cell are presented in this table. Medians, interquartile ranges (IQR), minima and maxima are given for all single-sorted cells (all), single-sorted p24+ cells, and single-sorted p24- cells.
