# Supplementary material for: Potent latency reversal by Tat RNA-containing nanoparticle enables multi-omic analysis of the HIV-1 reservoir
Source: Nat Commun. 2023 Dec 18;14:8397. doi: 10.1038/s41467-023-44020-5 (PMC10728105; doi:10.1038/s41467-023-44020-5)
Supplement: Supplementary file 3 — Description of Additional Supplementary Files [file 41467_2023_44020_MOESM3_ESM.pdf]

Title: Supplementary Data 1.
Description: Masterfile of the single-sorted p24+ cells for STIP-Seq analyses performed on 5 participants (MRC01, MRC04, MRC08, MRC15, STAR10). For each sorted cell, the table contains information about the phenotype (TCM/TTM/TEM/TTd), the mean fluorescence intensity for each p24 antibody (28B7-APC, KC57-FITC), the integration site, the list of all near full length PCRs that have been tested, and the proviral genome classification.

Title: Supplementary Data 2.
Description: Masterfile of the single-sorted cells for Smart-seq2 analyses performed on 7 participants (MRC01, MRC03, MRC04, MRC08, MRC15, MRC21, STAR10). For each sorted cell, the table contains information about the phenotype (TCM/TTM/TEM/TTd), the p24 status (NEG/POS), the stimulation performed (non-stimulated, Tat-LNP, Tat-LNP/PNB, PMA/i), the DNA quantitation using PicroGreen and the i5/i7 index sequences used for Illumina libraries. For each p24+ cell, information is provided about the integration site (inferred from the STIP-Seq sequences), the percentage of reads mapping to the HIV reference HXB2 and the proviral genome classification.

Title: Supplementary Data 3.
Description: (i) List of the differentially expressed genes (DEG) using linear regression models for microarray data analysis (Limma). (ii) Gene Set Enrichment Analysis (GSEA) to assess gene-set enrichment on the order ranked gene list using ClusterProfiler. Samples were collected after 6h of stimulation. Cluster 2 (Tat-LNP/PNB and PNB-stimulated cells) and cluster 3 (PMA/i-stimulated cells) were compared to cluster 1 (non-stimulated samples or treated with DMSO, HA-LNP, Tat-LNP).

Title: Supplementary Data 4.
Description: (i) List of the differentially expressed genes (DEG) using linear regression models for microarray data analysis (Limma). (ii) Gene Set Enrichment Analysis (GSEA) to assess gene-set enrichment on the order ranked gene list using ClusterProfiler. Samples were collected after 24h of stimulation. Cluster 2 (Tat-LNP/PNB and PNB-stimulated cells) and cluster 3 (PMA/i-stimulated cells) were compared to cluster 1 (non-stimulated samples or treated with DMSO, HA-LNP, Tat-LNP).

Title: Supplementary Data 5.
Description: List of the differentially expressed genes (DEG) between p24+ and p24- cells following Tat-LNP/PNB and Tat-LNP stimulation. Normalized read counts from the Smart-seq2 analyses were used to infer differentially expressed genes (DEG) using the MAST algorithm in R. P-values lower than 0.05 after Bonferroni corrections are considered significant.

Title: Supplementary Data 6.
Description: Gene Set Enrichment Analysis (GSEA) on the order ranked gene list (Supplementary data 5) using ClusterProfiler: gene sets differentially expressed between

p24+ and p24- cells following Tat-LNP stimulation. Gene sets with an adjusted p value below 0.05 are indicated in the table. Gene Ontology gene sets were downloaded from MsigD (GSEA_GO), while canonical pathway gene sets combine gene sets from the WikiPathways, Reactome, KEGG, PID and BioCarta databases (GSEA_canonical).
