# Supplementary material for: Potent latency reversal by Tat RNA-containing nanoparticle enables multi-omic analysis of the HIV-1 reservoir
Source: Nat Commun. 2023 Dec 18;14:8397. doi: 10.1038/s41467-023-44020-5 (PMC10728105; doi:10.1038/s41467-023-44020-5)
Supplement: Supplementary file 10 — Reporting Summary [file 41467_2023_44020_MOESM10_ESM.pdf]

nature portfolio

Corresponding author(s): Marion Pardons
Linos Vandekerckhove

Last updated by author(s): Nov 6, 2023

Reporting Summary

Nature Portfolio wishes to improve the reproducibility of the work that we publish. This form provides structure for consistency and transparency in reporting. For further information on Nature Portfolio policies, see our Editorial Policies and the Editorial Policy Checklist.

Statistics

For all statistical analyses, confirm that the following items are present in the figure legend, table legend, main text, or Methods section.

n/a	Confirmed	
☐	☒	The exact sample size (*n*) for each experimental group/condition, given as a discrete number and unit of measurement
☐	☒	A statement on whether measurements were taken from distinct samples or whether the same sample was measured repeatedly
☐	☒	The statistical test(s) used AND whether they are one- or two-sided<br>*Only common tests should be described solely by name; describe more complex techniques in the Methods section.*
☐	☒	A description of all covariates tested
☐	☒	A description of any assumptions or corrections, such as tests of normality and adjustment for multiple comparisons
☐	☒	A full description of the statistical parameters including central tendency (e.g. means) or other basic estimates (e.g. regression coefficient) AND variation (e.g. standard deviation) or associated estimates of uncertainty (e.g. confidence intervals)
☐	☒	For null hypothesis testing, the test statistic (e.g. *F*, *t*, *r*) with confidence intervals, effect sizes, degrees of freedom and *P* value noted<br>*Give P values as exact values whenever suitable.*
☒	☐	For Bayesian analysis, information on the choice of priors and Markov chain Monte Carlo settings
☒	☐	For hierarchical and complex designs, identification of the appropriate level for tests and full reporting of outcomes
☐	☒	Estimates of effect sizes (e.g. Cohen's *d*, Pearson's *r*), indicating how they were calculated

Our web collection on statistics for biologists contains articles on many of the points above.

Software and code

Policy information about availability of computer code

Data collection	Flow cytometry: BD FACSDiva software
Data analysis	Flow Cytometry Standard files (fcs) were analyzed using FlowJo v10.6.2. Index Sorted data were analyzed using the "index sort gating.fjs" script, freely available on the FlowJo Exchange site (http://exchange.flowjo.com). A step-by-step protocol for the analysis of index sorted data is provided here: https://flowjo.typepad.com/the_daily_dongle/2016/12/how-to-use-flowjos-script-editor-with-index-sorted-data.html.<br>Bar and line charts were generated with Graphpad Prism (v8.0.2). Violin plots were generated in R (v4.1.1) using the ggplot2 package (v3.3.5) and the “geom_sina” function from the ggforce package (v0.3.3). The correlation matrix on Supplementary Fig.5A was generated in R (v3.6.1) using the “dist” function from the stats package (v4.0.2), as well as the ComplexHeatmap package (v2.4.3). The gene ontology heatmap on Supplementary Fig.5B was generated in R (v3.6.1) using the ComplexHeatmap package (v2.4.3). The heatmap on Supplementary Fig.6B was generated in R (v4.2.0) using the dittoHeatmap function of the dittoSeq package (v1.8.1). The phylogenetic tree on Fig.4A was visualized in R (v4.1.1) using the ggtree (v3.2.1), ape (v5.6) and the phytools (v1.0-1) packages.

For manuscripts utilizing custom algorithms or software that are central to the research but not yet described in published literature, software must be made available to editors and reviewers. We strongly encourage code deposition in a community repository (e.g. GitHub). See the Nature Portfolio guidelines for submitting code & software for further information.

Data

Policy information about availability of data

All manuscripts must include a data availability statement. This statement should provide the following information, where applicable:

- Accession codes, unique identifiers, or web links for publicly available datasets
- A description of any restrictions on data availability
- For clinical datasets or third party data, please ensure that the statement adheres to our policy

HIV-1 near full-length sequences that support the findings of this study have been deposited in Genbank (accession codes: OQ596882-OQ596960).
Smart-seq2 data have been uploaded on SRA (accession code: PRJNA888069).
Microarray data have been uploaded on GEO (accession code: GSE247402)
Scripts concerning de novo assembly of HIV-1 genomes can be found at the following GitHub page: https://github.com/laulambr/virus_assembly.
Data obtained to generate all figures from this paper are included in the Source Data file.

Research involving human participants, their data, or biological material

Policy information about studies with human participants or human data. See also policy information about sex, gender (identity/presentation), and sexual orientation and race, ethnicity and racism.

Reporting on sex and gender	This study includes a total of of n = 23 HIV-1 seropositive individuals on stably suppressive ART (Supplementary Table 1). 2/23 individuals are female, 21/23 are male. The limited representation of female individuals in our study is a direct reflection of the infected population in Belgium, which predominantly consists of men who have sex with men.
Reporting on race, ethnicity, or other socially relevant groupings	Race/ethnicity/social groups are not reported in this study.<br>Most participants described in our study are infected with a subtype B virus (18/23) (Supplementary Table 1).
Population characteristics	- Median age: 50.3 years (IQR: 39.2-55.6)<br>- Median CD4/CD8 ratio: 0.8 (IQR: 0.7-1.0)<br>- Median NADIR (lowest CD4 count): 262.5 cells/mm3 (IQR: 179.8-376.8)<br>- Median ART duration: 8.7 years (IQR: 5.5 - 16.4)<br>All characteristics of the participants can be found in Supplementary Table 1.
Recruitment	People living with HIV on antiretroviral therapy were recruited following the inclusion criteria:<br>• Age = or >18 years and < 65 years<br>• CD4 count at screening > 350/µl<br>• Viral load < 40 copies/ml determined by CobasTaqMan HIV-1 test v2.0 assay for at least 2 years (one blip < 200 copies/ml is allowed)<br>• Ability and willingness to have blood and tissue samples collected and stored for 20 years and used for various research purposes
Ethics oversight	All participants were adults and signed informed consent forms approved by the Ethics Committee of the Ghent University Hospital

Note that full information on the approval of the study protocol must also be provided in the manuscript.

Field-specific reporting

Please select the one below that is the best fit for your research. If you are not sure, read the appropriate sections before making your selection.

[x] Life sciences   [ ] Behavioural & social sciences   [ ] Ecological, evolutionary & environmental sciences

For a reference copy of the document with all sections, see nature.com/documents/nr-reporting-summary-flat.pdf

Life sciences study design

All studies must disclose on these points even when the disclosure is negative.

Sample size	Sample size calculations were not performed due to the limited number of samples.
Data exclusions	No data were excluded from the analyses.
Replication	- To measure the frequency of p24+ cells by HIV-Flow following PMA/i and Tat-LNP/PNB (Fig.1D), 1-3 replicates were performed for each participant (the number of replicates for each individual is indicated in the Source Data file).<br>- Frequencies of p24+ cells measured by the HIV-Flow assay following Tat-LNP stimulation were reproducible across experiments (4-6 independent experiments, n = 4 individuals, mean coefficient of variation = 0.21; Supplementary Fig.1B).<br>- Experiments involving single-cell sorting (STIP-Seq, Smart-seq2) were performed one or two times for each participant, in order to obtain a minimum of 10 p24+ cells per participant and per condition. Similar frequencies of p24+ cells were obtained between replicates, and identical

clones of HIV-infected cells could be retrieved between replicates.
- For Fig 8D-E (ASO treatment of CD4 T cells prior to in vitro HIV infection): the experiment was repeated on three HIV negative donors. qPCR was done in duplicates.
- For Fig 8F-G (ASO treatment of latently infected SupT1 cell line prior Tat-LNP stimulation): two independant experiments were performed. Each condition was tested in duplicates. qPCR was done in duplicates.

Randomization	Randomization is not applicable. This is a single-arm study: All participants are people living with HIV on antiretroviral therapy.
Blinding	Blinding is not applicable. This is a single-arm study: All participants are people living with HIV on antiretroviral therapy. There was no experimental drug administration performed.

Reporting for specific materials, systems and methods

We require information from authors about some types of materials, experimental systems and methods used in many studies. Here, indicate whether each material, system or method listed is relevant to your study. If you are not sure if a list item applies to your research, read the appropriate section before selecting a response.

Materials & experimental systems

n/a	Involved in the study	
☐	☒	Antibodies
☒	☐	Eukaryotic cell lines
☒	☐	Palaeontology and archaeology
☒	☐	Animals and other organisms
☐	☒	Clinical data
☒	☐	Dual use research of concern
☒	☐	Plants

Methods

n/a	Involved in the study	
☒	☐	ChIP-seq
☐	☒	Flow cytometry
☒	☐	MRI-based neuroimaging

Antibodies

Antibodies used	Fixable Viability Stain 510 was obtained from ThermoFisher Scientific (#L34957, 1/1000). The following antibodies were used in staining experiments: CD3 AF700 Clone UCHT1 (BD Biosciences, #557943, 1/50), CD4 Pe-Cy7 Clone L200 (BD Bioscience, #560644, 1/200) and CD4 BV786 Clone SK3 (BD Biosciences, #563881, 1/200), CD8 BV510 Clone RPA-T8 (BioLegend, #301047, 1/200), CD45RO BV421 Clone UCHL1 (BD Biosciences, #562649, 1/100) or CD45RO PE Clone HI100 (BD Biosciences, #5555493, 1/20), CD27 BV605 Clone L128 (BD Biosciences, #562656, 1/100), PD1 BB700 Clone EH12.1 (BD Biosciences, #566461, 1/100), CD69 PerCP-Cy5.5 Clone FN50 (BD Biosciences, #560738) and CD69 APC (BioLegend, #310909, 1/50), CD25 BV421 Clone M-A251 (BD Biosciences, #562443, 1/50), HLA-DR FITC Clone REA805 (Miltenyi Biotech, #130-111-788, 1/50), GZMA PeCy7 Clone CB9 (BioLegend, #507221, 1/25), GZMB PB Clone GB11 (BioLegend, #515407, 1/50), CCL5 PerCP/Cyanine5.5 Clone VL1 (BioLegend, #515507, 1/50), CD127 PE-CF594 Clone HIL-7R-M21 (BD Biosciences, #562397, 1/50). For p24 staining, we used a combination of two antibodies: p24 KC57-FITC (Beckman Coulter, #6604665, 1/500) and p24 28B7-APC (MediMabs, #MM-0289-APC, 1/400).
Validation	- All antibodies are commercially available and were bought from BD Biosciences, BioLegend, and ThermoFisher Scientific. Titrations were performed for all antibodies to determine optimal antibody concentrations and dilutions used are reported in the manuscript and in the reporting summary.<br>- p24 KC57-FITC (Beckman Coulter): the certificate of analysis can be found on the Beckman Coulter website. It is certified that each batch of p24 KC57 meets the requirements for flow cytometry experiments. Clone description: The KC57 antibody identifies the 55, 39, 33 and 24 kDa proteins of the core antigens of the human immunodeficiency virus type 1 (HIV-1). The 55 kDa protein is the precursor protein for the core antigen. The 39 and 33 kDa proteins are intermediate products and the 24 kDa protein is the core protein.<br>- p24 28B7-APC (MediMabs): This antibody was generated from HIV-infected individuals by a method based on the Epstein-Barr virus transformation of peripheral blood mononuclear cells (PBMCs) followed by fusion with heteromyeloma cells. The epitope for this antibody is discontinuous. The antibody recognizes the p24 core or capsid protein of HIV-1. Specific for: HIV-1<br>References: p24 KC57 and 28B7 were used and validated by Pardons et al, Plos Pathogens 2019; Cole et al, Nature Commun. 2021; Gantner et al, Nature Commun. 2020; Gantner et al, Immunity 2023; Dufour et al, Nature Commun. 2023

Clinical data

Policy information about clinical studies

All manuscripts should comply with the ICMJE guidelines for publication of clinical research and a completed CONSORT checklist must be included with all submissions.

Clinical trial registration	NCT04305665, NCT02641756, NCT02590354
Study protocol	https://clinicaltrials.gov/study/NCT04305665<br>https://clinicaltrials.gov/study/NCT02641756<br>https://clinicaltrials.gov/study/NCT02590354
Data collection	NCT04305665 (Mercuri: MRC): June 2020 - December 2025 (recruitment still ongoing; Ghent University Hospital). The Mercuri participants were recruited specifically for the present study.<br>NCT02641756 (STAR): April 2016 - August 2019 (recruitment completed; Ghent University Hospital)<br>NCT02590354 (ISALA): January 2016 - June 2018 (recruitment completed; Ghent University Hospital, Institute of Tropical Medicine, Saint-Pierre University Hospital, Brussels University Hospital)<br>The two participants from the STAR and ISALA studies were not recruited in the context of this study. These two participants exhibited elevated frequencies of p24+ cells upon stimulation. Their inclusion further expanded the pool of participants for our investigations (e.g. phenotype of p24+ cells and STIP-Seq/Smart-seq2 analyses).<br>Written informed consents from all participants were obtained for the use of samples and data for this present work. Analyses presented in this manuscript are covered by the three protocols.
Outcomes	Primary outcome: using a multi-faceted approach to extensively study the HIV-1 latent reservoir. In this study, we used several assays to extensively characterize the translation-competent HIV-1 reservoir: measurement of the frequency of p24+ cells by HIV-Flow (described by Pardons et al, Plos Pathogens 2019); simultaneous assessment of the HIV integration site and near-full length HIV sequence from p24+ cells by STIP-Seq (described by Cole et al, Nature Commun. 2021); transcriptomic analysis of p24+ cells by Smart-seq2 (described by Picelli et al, Nature Protocols 2014).<br>Secondary outcome: NA

Flow Cytometry

Plots

Confirm that:

- [x] The axis labels state the marker and fluorochrome used (e.g. CD4-FITC).
- [x] The axis scales are clearly visible. Include numbers along axes only for bottom left plot of group (a 'group' is an analysis of identical markers).
- [x] All plots are contour plots with outliers or pseudocolor plots.
- [x] A numerical value for number of cells or percentage (with statistics) is provided.

Methodology

Sample preparation	CD4 T cells were isolated from PBMC by negative magnetic selection using the EasySep Human CD4 T Cell Enrichment Kit (StemCell Technology, #19052). Purity was typically >98%. CD4 T cells were resuspended at 2x106 cells/mL in RPMI + 10% Fetal Bovine Serum and antiretroviral drugs were added to the culture (200 nM raltegravir, 200 nM lamivudine) to avoid new cycles of replication. Cells were rested for at least an hour at 37 °C before being stimulated with the following LRAs: 1 µg/mL ionomycin (Sigma, #I9657) and 162 nM PMA (Sigma, #P8139), 50 nM panobinostat (Selleckchem, LBH589), Tat-LNP (250 ng/mL; 1.4 nM). When combined, the same concentrations were used for Tat-LNP and PNB. Cells were stimulated for 24 or 48h, depending on the stimulation condition.
Instrument	Flow cytometry: BD LSRFortessa, BD FACSAria Fusion
Software	Data collection: BD FACSDiva software<br>Data analysis: FlowJo v10.6.2
Cell population abundance	The frequency of p24+ cells ranged between 1 and 50 per million CD4 T cells.<br>As shown in Supplementary Figure 4A, no HIV reads were detected in single-cell sorted p24- cells, while HIV reads were detected in almost all single-cell sorted p24+ cells, confirming the post-sort purity of those two populations.
Gating strategy	For HIV-Flow experiments: CD4 T cells from an HIV-negative control or a non-stimulated sample from the same participant were included to set the threshold of positivity.<br>Gating strategies can be found in the Supplementary Figures (Sup Fig.1A: HIV-Flow, Sup Fig.2: CD3/CD4, CD45RO/CD27 (subsets of CD4 T cells), Sup Fig.7 (GZMA, GZMB, IL7R, CCL5).

- [x] Tick this box to confirm that a figure exemplifying the gating strategy is provided in the Supplementary Information.
